# Hospitalization Pattern for Chronic Lower Respiratory Diseases in Australia: A Retrospective Ecological Study

**DOI:** 10.7759/cureus.33162

**Published:** 2022-12-31

**Authors:** Mohammad S Dairi

**Affiliations:** 1 Department of Internal Medicine, Faculty of Medicine, Umm Al-Qura University, Makkah, SAU

**Keywords:** respiratory diseases, asthma, copd, australia, admission

## Abstract

Background: Chronic lower respiratory diseases are among the commonest causes of hospital admission worldwide. Identifying the trends in hospital admission due to chronic lower respiratory diseases is important for public health and policy makers.

Aim: The aim of this study was to examine the hospitalization profile related to chronic lower respiratory diseases in Australia during the past 21 years.

Method: A retrospective ecological study was conducted using hospital admission data taken from the National Hospital Morbidity Database (NHMD). Hospital admissions data for chronic lower respiratory diseases were extracted for the period between 1998 and 2019. The Pearson Chi-square test for independence was used to estimate the variation in hospital admission rates.

Results: The hospitalization rate for chronic lower respiratory diseases rate decreased by 14.4%, from 568.90 (95%CI 565.50-572.30) in 1998 to 486.95 (95%CI 484.24-489.66) in 2019 per 100,000 persons, p<0.05. Rates of same-day hospitalization for chronic lower respiratory diseases increased by 62.7% from 1998 to 2019, while rates of overnight-stay hospital admission for chronic lower respiratory diseases decreased by 23.7% from 1998 to 2019. During the study duration, the hospitalization rates for bronchiectasis and other chronic obstructive pulmonary disease increased by 120.0% and 34.7%, respectively. The hospital admissions rates for emphysema, status asthmaticus, simple and mucopurulent chronic bronchitis, bronchitis, not specified as acute or chronic, unspecified chronic bronchitis, and asthma decreased by 94.8%, 92.6%, 70.7%, 66.3%, 46.0%, and 32.3%, respectively. The rates of hospitalization among patients aged 75 years and above increased by 3.9%, while younger age groups including those aged younger than 15 years, 15-59 years, and 60-74 years showed a reduction in the rate of hospitalization by 53%, 22.8%, and 19.7%, respectively.

Conclusion: Overall, the hospitalization rate for chronic lower respiratory diseases is seen to have decreased over the study period. Patients with chronic obstructive pulmonary disease (COPD) and the elderly group have a higher rate of hospitalization. Future studies are needed to investigate factors associated with the increase in the rate of hospitalization among the elderly age group.

## Introduction

Chronic lower respiratory diseases are a set of disorders that affect the lung [1], including bronchitis, not specified as acute or chronic, simple and mucopurulent chronic bronchitis, unspecified chronic bronchitis, emphysema, other chronic obstructive pulmonary disease, asthma, status asthmatics, and bronchiectasis [2]. Chronic obstructive pulmonary disease (COPD) and asthma are two of the most common chronic lower respiratory diseases [3,4]. Chronic lower respiratory disorders are a significant reason for mortality and morbidity [1]. The third leading cause of death worldwide is COPD, which is the worst form of chronic lower respiratory disease [5]. In Australia, COPD accounted for 3.9% of all deaths, with 6,311 deaths, ranking as the fifth-leading cause of death in 2020 [6]. Older people are significantly more susceptible to COPD, and the disease's mortality and morbidity rapidly increase with age [7]. In addition, respiratory disorders are the most common postoperative complications in older patients [8].

In Australia, chronic respiratory diseases affect 31% of the population. Around 2.7 million Australians, or 11% of the 7.4 million people with chronic respiratory diseases, had asthma [9,10]. In Australia, 375,800 individuals (1.5% of the population) had COPD in 2020-2021 [9]. Asthma and COPD are ranked tenth and fourth in Australia, respectively, for the overall burden of disease [11]. Furthermore, among children aged 5-14 years, the disease burden is primarily due to asthma [10,11]. Also, it is becoming more widely recognized that COPD and asthma can co-exist [11].

Patients with chronic respiratory diseases that require hospitalization have severe symptoms that cannot be controlled by a general practitioner or at home [10]. There were 70,951 COPD episodes involving hospital admission in Australia in 2019/2020 among people aged 45 years and above [10]. In the same period, patients of all ages had 32,822 episodes of asthma that required hospital admission [10]. Recently, Naser and colleagues explored the hospitalization profile for different healthcare conditions including respiratory diseases in the United Kingdom and reported high rates of hospitalization among different study populations [12-24]. Understanding patterns in chronic lower respiratory disease hospitalization will aid medical professionals in managing these conditions more efficiently, which in turn helps to minimize the number of hospitalization related to these diseases. Therefore, the aim of this study was to examine the hospitalization profile related to chronic lower respiratory diseases in Australia during the past 21 years.

## Materials and methods

Study design

This was a retrospective ecological study that examined the hospitalization profile for chronic lower respiratory diseases in Australia between 1998 and 2019.

Data sources

National Hospital Morbidity Database

The National Hospital Morbidity Database (NHMD) is part of the National Hospitals Data Collection, which includes the major national hospital databases held by the Australian Institute of Health and Welfare (AIHW) [25]. NHMD is an online database comprising data provided by state and territory health authorities in Australia [26]. Data from morbidity data collection systems of patients admitted to private and public hospitals in Australia are gathered at the NHMD as sets of episode-level records. The information provided is based on the National Minimum Data Set (NMDS) for admitted patient care and includes information on the patients' diagnoses, external causes of injury and poisoning, length of hospital stays, administrative issues, and procedures they underwent. It also includes demographic information. The objective of the NMDS for admitted patient care is the collecting of information regarding the care given to hospitalized patients in Australian hospitals. The NMDS covers episodes of care for patients who have been admitted to hospitals from all alcohol and drug treatment facilities, independent day hospitals, and private and public psychiatric and acute hospitals. Hospitalization episodes related to all chronic lower respiratory diseases were identified using the following International Classification of Diseases (ICD) codes (J40-J47).

Australian Bureau of Statistics (ABS)

Mid-year population data were collected from the ABS from 1998 to 2019 [27]. The historical population was used to collect population data between 1998 and 2016 [28]. National, state, and territory population was used to collect population data between 2017 and 2019 [29].

Study population

This study collected data on all private and public hospitalizations for chronic lower respiratory diseases in Australia from 1998 to 2019 [30].

Statistical analysis

IBM SPSS Statistics for Windows, Version 27.0 (Released 2020; IBM Corp., Armonk, New York, United States) was used for all analyses. Using the chronic lower respiratory disease episodes divided by the mid-year population, hospitalization rates with 95% confidence intervals (CIs) were determined. The Pearson chi-square test for independence was used to estimate the variation in hospitalization rates between 1998 and 2021 because we were using independent frequency data with a sufficient sample size.

## Results

Trends in total chronic lower respiratory diseases hospitalization

A total of 2,250,127 chronic lower respiratory disease hospitalization episodes were recorded in Australia between 1998 and 2019. The total annual number of chronic lower respiratory diseases hospitalization for various reasons increased by 15.4% from 107,023 in 1998 to 123,518 in 2019. However, the hospitalization rate decreased by 14.4%, from 568.90 (95%CI 565.50-572.30) in 1998 to 486.95 (95%CI 484.24-489.66) in 2019 per 100,000 persons, p<0.05.

**Figure 1:**
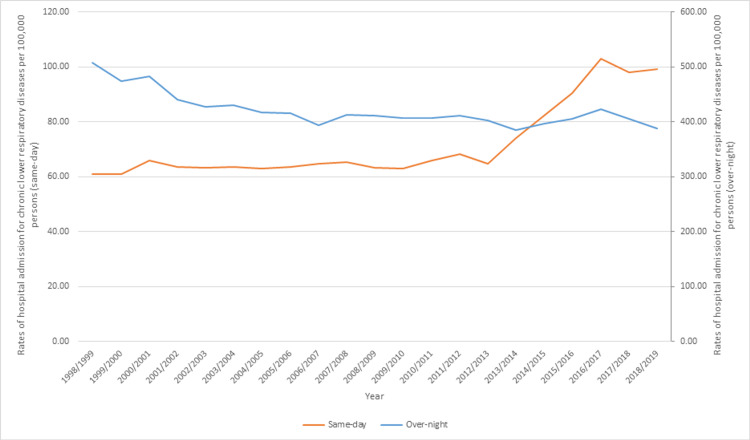
Rates of same-day and overnight-stay hospital admission for chronic lower respiratory diseases in Australia between 1998 and 2019.

Overnight-stay hospital admission accounted for 85.2% of the total number of chronic lower respiratory diseases hospitalization, and 14.8% were same-day patients. Rates of same-day hospitalization increased by 62.7%, from 60.92 (95%CI 59.80-62.03) in 1998 to 99.10 (95%CI 97.88-100.33) in 2019 per 100,000 persons. Rates of overnight-stay hospital admission decreased by 23.7%, from 507.98 (95%CI 504.77-511.20) in 1998 to 387.48 (95%CI 385.06-389.89) in 2019 per 100,000 persons (Figure 1).

Other COPD was the most common chronic lower respiratory disease hospitalization reasons accounting for 54.5% of the total number of chronic lower respiratory diseases hospitalization, followed by asthma with 32.8% (Table 1).

**Table 1:** Percentage of chronic lower respiratory diseases hospital admission from total number of episodes. ICD: International Classification of Diseases

ICD code	Description	Percentage from total number of admissions
J40	Bronchitis, not specified as acute or chronic	2.3%
J41	Simple and mucopurulent chronic bronchitis	<0.1%
J42	Unspecified chronic bronchitis	0.4%
J43	Emphysema	0.8%
J44	Other chronic obstructive pulmonary disease (with acute lower respiratory infection and with acute exacerbation)	54.5%
J45	Asthma	32.8%
J46	Status asthmaticus	4.5%
J47	Bronchiectasis	4.4%

Trends in types of chronic lower respiratory diseases hospitalization (based on separations)

**Figure 2:**
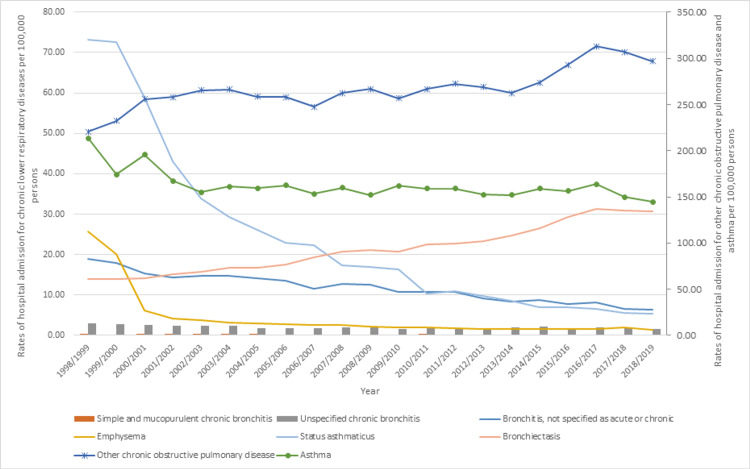
Rates of hospitalization in Australia from 1998 to 2019, stratified by type.

During the study period, the hospitalization rate for bronchiectasis and other COPD increased by 120.0% and 34.7%, respectively. On the other hand, the hospital admission rates for emphysema, status asthmaticus, simple and mucopurulent chronic bronchitis, bronchitis, not specified as acute or chronic, unspecified chronic bronchitis, and asthma decreased by 94.8%, 92.6%, 70.7%, 66.3%, 46.0%, and 32.3%, respectively (Table 2, Figure 2).

**Table 2:** Percentage change in the hospital admission rates

Diseases	Rate of diseases in 1998 per 100,000 persons (95% CI)	Rate of diseases in 2019 per 100,000 persons (95% CI)	Percentage change from 1998–2019
Bronchitis, not specified as acute or chronic	18.87 (18.25 – 19.49)	6.36 (6.04 – 6.67)	-66.3%
Simple and mucopurulent chronic bronchitis	0.40 (0.31 – 0.49)	0.12 (0.08 – 0.16)	-70.7%
Unspecified chronic bronchitis	3.01 (2.77 – 3.26)	1.63 (1.47 – 1.79)	-46.0%
Emphysema	25.66 (24.94 – 26.39)	1.32 (1.18 – 1.47)	-94.8%
Other chronic obstructive pulmonary disease	220.41 (218.29 – 222.53)	296.85 (294.73 – 298.96)	34.7%
Asthma	213.39 (211.30 – 215.47)	144.46 (142.98 – 145.94)	-32.3%
Status asthmaticus	73.17 (71.94 – 74.39)	5.44 (5.15 – 5.72)	-92.6%
Bronchiectasis	13.99 (13.45 – 14.52)	30.78 (30.10 – 31.46)	120.1%

Trends in total chronic lower respiratory diseases hospitalization (based on gender)

**Figure 3:**
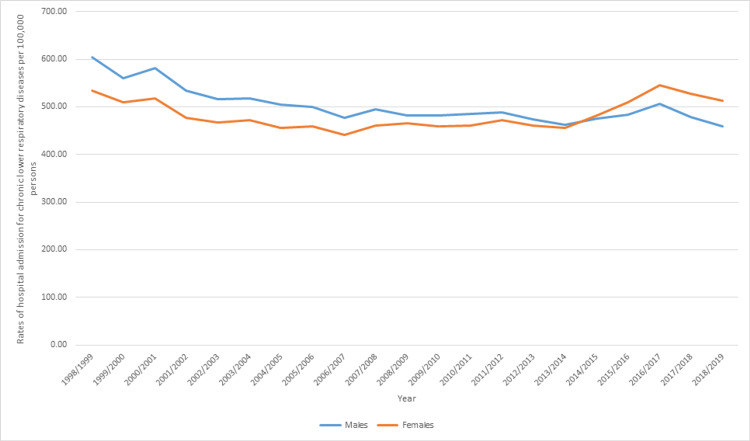
Rates of hospital admission in Australia, stratified by gender.

Males contributed to 50.5% of the total number of chronic lower respiratory diseases hospitalization accounting for 1,136,844 episodes with a mean of 54,135 episodes per year. Chronic lower respiratory diseases hospitalization rate among males decreased by 24.0%, from 604.73 (95%CI 599.75-609.70) in 1998 to 459.76 (95%CI 456.02-463.50) in 2019 per 100,000 persons. The hospitalization rate among females decreased by 3.9%, from 533.57 (95%CI 528.94-538.21) in 1998 to 512.94 (95%CI 509.03-516.86) in 2019 per 100,000 persons (Figure 3).

Trends in chronic lower respiratory diseases hospitalization (based on age group)

**Figure 4:**
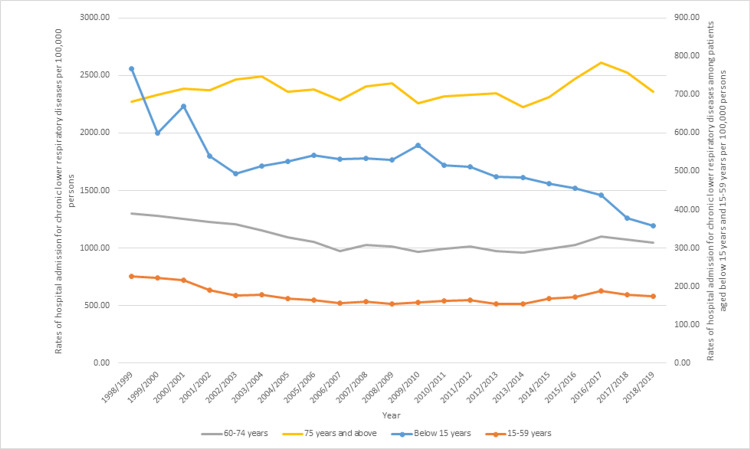
Rates of hospital admission in Australia, stratified by age group.

Regarding age group diversity, the age group of 75 years and above accounted for 30.3% of the total number of chronic lower respiratory diseases hospitalization, followed by the age group 60-74 years with 27.5%, the age group 15-59 years with 21.9%, and then the age group of 15 years and below with 20.3%. The rate of hospitalization for chronic lower respiratory diseases among patients aged below 15 years decreased by 53.3%, from 767.50 (95%CI 758.87-776.12) in 1998 to 358.11 (95%CI 352.74-363.49) in 2019 per 100,000 persons. The rate of hospitalization for chronic lower respiratory diseases among patients aged 15-59 years decreased by 22.8%, from 226.94 (95%CI 224.23-229.66) in 1998 to 175.10 (95%CI 172.99-177.20) in 2019 per 100,000 persons. The rate of hospitalization for chronic lower respiratory diseases among patients aged 60-74 years decreased by 19.7%, from 1,301.96 (95%CI 1,286.50-1,317.43) in 1998 to 1,044.91 (95%CI 1,034.52-1,055.30) in 2019 per 100,000 persons. The rate of hospitalization for chronic lower respiratory diseases among patients aged 75 years and above increased by 3.9%, from 2,271.90 (95%CI 2,243.01-2,300.80) in 1998 to 2,360.48 (95%CI 2,338.01-2,382.94) in 2019 per 100,000 persons (Figure 4).

Trends in chronic lower respiratory diseases hospitalization (based on age group and gender)

Below 15 years

During the study period, chronic lower respiratory disease hospitalization rates among patients aged 0-4 years, 5-9 years, and 10-14 years decreased by 63.9%, 31.4%, and 52.4%, respectively. Chronic lower respiratory disease hospitalization rates among male patients aged 0-4 years, 5-9 years, and 10-14 years decreased by 63.9%, 29.5%, and 51.9%, respectively. Chronic lower respiratory disease hospitalization rates among female patients aged 0-4 years, 5-9 years, and 10-14 years decreased by 63.8%, 34.3%, and 53.1%, respectively.

Age Group of 15 - 59 years

During the study period, the hospitalization rates among patients aged 15-19 years, 20-24 years, 25-29 years, 30-34 years, 35-39 years, 40-44 years, 45-49 years, 50-54 years, and 55-59 years decreased by 49.1%, 59.0%, 52.9%, 39.5%, 16.7%, 9.0%, 3.3%, 5.3%, and 14.8%, respectively. Hospitalization rates among male patients aged 15-19 years, 20-24 years, 25-29 years, 30-34 years, 35-39 years, 40-44 years, 50-54 years, and 55-59 years decreased by 48.7%, 64.6%, 56.1%, 43.5%, 6.6%, 21.0%, 3.7%, and 17.0%, respectively. Hospitalization rates among male patients aged 45-49 years increased by 9.0%. Hospitalization rates among female patients aged 15-19 years, 20-24 years, 25-29 years, 30-34 years, 35-39 years, 40-44 years, 45-49 years, 50-54 years, and 55-59 years decreased by 49.2%, 55.4%, 51.1%, 37.8%, 21.2%, 2.6%, 9.6%, 7.4%, and 14.1%, respectively.

Age Group of 60-74 years

During the study period, hospitalization rates among patients aged 60-64 years, 65-69 years, and 70-74 years decreased by 20.4%, 20.9%, and 16.6%, respectively. Hospitalization rates among male patients aged 60-64 years, 65-69 years, and 70-74 years decreased by 31.9%, 34.2%, and 35.1%, respectively. Hospitalization rates among female patients aged 60-64 years and 65-69 years decreased by 8.5% and 5.3%, respectively. Hospitalization rates among female patients aged 70-74 years increased by 9.0%.

Above 74 years

During the study period, hospitalization rates among patients aged 75-79 years and 80-84 years decreased by 7.4% and 0.8%, respectively. Hospitalization rates among patients aged 85 years and over increased by 32.3%. Hospitalization rates among male patients aged 75-79 years, 80-84 years, and 85 years and over decreased by 29.3%, 23.9%, and 2.2%, respectively. Hospitalization rates among female patients aged 75-79 years, 80-84 years, and 85 years and over increased by 21.3%, 24.4%, and 59.8%, respectively.

## Discussion

In this ecological study, the hospitalization profile related to chronic lower respiratory diseases in Australia was explored over a 21-year period of time. Respiratory diseases, including chronic lower respiratory diseases, have a considerably high prevalence rate in Australia [31]. In addition, chronic lower respiratory diseases are among the top leading causes of death in Australia [32]. However, according to the Australian Institute of Health and Welfare, the death rate and hospitalization rate of chronic lower respiratory diseases have decreased in the last couple of years [31]. Similarly, previous studies across the world, including North and South America and Europe, have reported a negative trend in hospitalization due to asthma and COPD. This was reflected in this study, which showed that the hospitalization rate for chronic lower respiratory diseases decreased by 14.4%, which was also consistent with previous studies worldwide [33-37]. Evidence-based regional, national, and international guidelines for asthma management have been implemented in recent years [38-40]. In addition, better diagnostic methods and effective medications are now available for the management of asthma and COPD [41]. Therefore, these factors may have played an important role in the decline in the hospitalization rate observed in this study. In addition, healthcare providers play an important role in the management and care of chronic lower respiratory disease exacerbations at the primary care level, which may have also contributed to reducing the burden on emergency rooms and hospital visits [42-44]. This was highlighted in this study, which showed that the rates of same-day hospitalization for chronic lower respiratory diseases increased by 62.7% from the year of 1998 to 2019, while the rates of overnight-stay hospital admission for chronic lower respiratory diseases decreased by 23.7%.

In the past years, the prevalence of COPD and asthma has increased significantly in Australia, with around 11% of the total population diagnosed with asthma in 2018, and around 8% of those older than 40 years of age diagnosed with COPD [45-47]. Asthma and COPD are among the most common respiratory diseases, which is consistent with this study that highlighted that COPD accounted for the most common hospitalization cases, followed by asthma and bronchiectasis.

During the study period, the hospitalization rate for COPD increased by 34.7%. However, the hospital admission rate for emphysema, status asthmaticus, simple and mucopurulent chronic bronchitis, bronchitis, not specified as acute or chronic, unspecified chronic bronchitis, and asthma decreased by 94.8%, 92.6%, 70.7%, 66.3%, 46.0%, and 32.3%, respectively. These results are consistent with the literature, which showed that the management of asthma and the incidence of acute asthma attacks have improved significantly in the past few years [43]. On the other hand, the upward trend of COPD hospitalization can be attributed to the aging population, knowing that COPD commonly affects the elderly group [48]. This was also reflected in the stratification analysis by age group in this study, as the rates of hospitalization for chronic lower respiratory diseases among patients aged 75 years and above increased by 3.9%, while younger age groups, including those aged younger than 15 years, 15-59 years, and 60-74 years showed a reduced rate of hospitalization with 53%, 22.8%, and 19.7%, respectively.

Age is an important factor in the risk of hospitalization and mortality of chronic respiratory diseases [49]. Patients suffering from COPD are likely to be diagnosed with more than one comorbid disease. Comorbidities such as cardiac diseases and diabetes are linked to both COPD and an increased risk of hospitalization [49]. These factors may explain the results shown in this study and that the trend of hospitalization has increased among the elderly age group. This is also consistent with a previous study in the UK, which concluded that hospital admissions due to diseases of the respiratory system are higher among the elderly age group [50].

In this study, there was no significant difference in the rate of hospitalization between males and females. However, in the last years of the study period, females had a higher percentage of hospitalizations due to chronic lower respiratory diseases compared to males. Recent data from the literature show an increase in the incidence of COPD among females and that COPD is no longer a disease of males [51]. In addition, previous studies in the UK showed that the rates of admissions due to chronic lower respiratory diseases in the UK were higher among females compared to males [50,52], which was consistent with the results of this study. Females tend to be more susceptible to smoking. In addition, females tend to have a longer life expectancy, which puts them at a higher risk of developing chronic lung diseases [51]. These factors may explain the increase in the rate of hospitalization due to chronic lower respiratory diseases among females compared to males. However, future studies directed at investigating the factors associated with the increase in female hospitalization due to chronic respiratory disease are needed.

This study has several advantages, including the fact that it is the first study to examine the hospitalization rate of chronic lower respiratory diseases in Australia over 21 years. In addition, in this study, several stratifications were conducted to highlight the differences among age groups and gender. However, this study has some limitations. First, this study used an ecological study design on the population level, and therefore, it is difficult to individualize the results on the individual patient level, which restricted the ability to identify important confounding variables. In addition, this study lacked important data on factors such as physical inactivity, comorbidities, and medication history, which may have impacted the rates of admission directly or indirectly.

## Conclusions

Overall, the rate of hospitalization for chronic lower respiratory diseases has decreased over the study period. However, patients with COPD and the elderly group have a higher rate of hospitalization. Future studies are warranted to investigate factors associated with the increase in the rate of hospitalization among the elderly age group.
